# NSAID-Induced Enteropathy in Rheumatoid Arthritis Patients with Chronic Occult Gastrointestinal Bleeding: A Prospective Capsule Endoscopy Study

**DOI:** 10.1155/2013/268382

**Published:** 2013-12-07

**Authors:** Ilja Tachecí, Petr Bradna, Tomáš Douda, Drahomíra Baštecká, Marcela Kopáčová, Stanislav Rejchrt, Jan Bureš

**Affiliations:** Second Department of Internal Medicine-Gastroenterology, Charles University in Prague, Faculty of Medicine in Hradec Králové and University Hospital, Sokolská 581, 500 05 Hradec Králové, Czech Republic

## Abstract

*Background*. The purpose of study was to evaluate the diagnostic yield of capsule endoscopy for NSAID-induced enteropathy and clinical, laboratory, and endoscopic characteristics of disease in patients with rheumatoid arthritis. *Methods*. 37 rheumatoid arthritis patients (30 women; mean age 55) treated with NSAIDs (>1 month), presented with anaemia and/or positive faecal occult blood testing, entered the study and underwent capsule endoscopy (EndoCapsule; Olympus), laboratory tests, and filled in questionnaires. *Results*. The prevalence of NSAID-induced enteropathy diagnosed by capsule endoscopy was 68% (25/37), classified as mild (red spots or erosions) in 18 (49%), moderate (10–20 erosions) in 4 (11%), and severe enteropathy (>20 erosions or ulcers) in 3 (8%) patients. We did not find statistically significant relationship between the enteropathy and gender, age, haemoglobin, leukocytes, albumin and CRP, or dyspepsia. The difference between subgroups of NSAIDs according to the COX specificity was not statistically significant. *Conclusions*. Capsule endoscopy is a highly accurate noninvasive method for evaluation of NSAID-induced enteropathy. It was revealed in a substantial section of the patients with rheumatoid arthritis and occult gastrointestinal bleeding, mostly classified as mild damage. No simple clinical or laboratory markers of the presence or severity of NSAID-induced enteropathy were recognised. This trial is registered with DRKS00004940.

## 1. Introduction 

Non-steroidal anti-inflammatory drugs (NSAIDs) are some of the most frequently prescribed medications in clinical practice for their analgesic, antipyretic, and anti-inflammatory effects, especially in rheumatic and other musculoskeletal disorders.

The adverse effects of NSAIDs therapy are well recognised and described especially in the gastroduodenal area [1, 2]; however, the prevalence of small intestinal structural or functional abnormalities induced by NSAIDs can be much higher. The first description of NSAID (aspirin)-induced gastropathy identified by endoscopy was presented by Douthwaite and Lintott in 1938 [3]. Small bowel damage due to indomethacin was observed for the first time in humans in the 70s [4].

The complete NSAIDs mechanism of action is not still fully understood, but it is at least partially based on an inhibition of cyclooxygenase (COX) [5]. The pathogenesis of enteropathy was initially thought to be associated with COX-1 inhibition only. However, it has been proven that selective COX-1 inhibition (or absence) does not lead to gastrointestinal lesions, and selective COX-2 inhibition (or absence) leads to ileocaecal mucosa damage, different from “classical” NSAID-induced enteropathy [6, 7]. Small bowel injury is induced by a combination of COX-1 inhibition with restricted mucosal blood flow and COX-2 inhibition probably through an unknown immunological effect. All systemic and local pathogenetic mechanisms lead, according to inflammation intensity, to erythema, erosions, and ulcers. The extensive fibroproduction during healing can cause strictures.

The spectrum of small intestinal side effects is wide and its clinical importance varies. This includes increased intestinal permeability [8, 9], ulcerations [10], perforation [11], diaphragm-like strictures [12, 13], and gastrointestinal bleeding [14]. Small bowel ulcers were observed in 8% of patients treated with NSAIDs in comparison with 0.6% of controls (with no history of NSAID use) in a prospective, autopsy-based study (713 cases) [15]. Initial endoscopy data were drawn from sonde enteroscopy and were not published until the early 90s [16]. The situation changed in the year 2000 when effective mini-invasive enteropathy diagnostics by means of wireless capsule endoscopy became widely available. The first video capsule endoscopy studies showed the wide range (6–78%) of enteropathy in healthy volunteers taking NSAIDs and proton pump inhibitors during a 2-week period or patients taking NSAID for rheumatoid arthritis or osteoarthritis [17–21].

There are still many questions about the real incidence of NSAID-induced enteropathy in different groups of patients, the spectrum of endoscopy findings in those patients according to the enteropathy severity, risk factors, leading clinical or laboratory findings, prevention, and treatment.

The objective of our prospective single-centre study is to answer some of them (including the diagnostic yield of capsule endoscopy) in a group of high-risk patients with rheumatoid arthritis (with anaemia and/or positive faecal occult blood testing (FOBT)) examined by means of wireless capsule endoscopy.

## 2. Materials and Methods

This was a prospective, endoscopist-blinded, capsule endoscopy study. A total of 37 patients (30 women; mean age 55, median 56 years and 7 males; mean age 55, median 56 years) were enrolled in study. The inclusion criteria were adult age (>18 years), rheumatoid arthritis, long-term NSAID therapy (>1 month), and anaemia (Hb < 135 g/L in men and <120 g/L in women) or positive FOBT (Hemoccult test, Beckman Coulter, Inc., USA) without signs of overt gastrointestinal bleeding. This subgroup of rheumatoid arthritis patients was chosen due to the expected higher prevalence of NSAID-induced damage to the small intestine in patients with suspected occult GI bleeding.

Patients with gastroesophageal reflux disease, gastroduodenal ulcers, small bowel diseases, inflammatory bowel disease, small intestinal stenosis, and pregnancy were excluded. Written informed consent was obtained from all. The study was approved by the Ethics Committee of University Teaching Hospital, Hradec Králové, Czech Republic.

All patients included in our study underwent capsule endoscopy and completed clinical investigation and blood tests (blood count, reticulocytes, Coombs test, serum iron level, iron binding capacity, ferritin, albumin, prealbumin, CRP, and erythrocyte sedimentation rate). Patients completed a questionnaire focused on the important anamnestic data.

### 2.1. Capsule Endoscopy

The methods of wireless capsule endoscopy used in our study were in full compliance with the Czech Society of Gastroenterology [22] and European Society of Gastrointestinal Endoscopy guidelines [23]. Capsule endoscopy was performed using the Olympus system (EndoCapsule). Patients were instructed not to use medication limiting small bowel visibility (iron supplements, pills with micropellets, etc.) for one week and fasted for 12 hours before swallowing the capsule. Fluids were allowed 2 hours later, followed by a light meal 4 hours later. Oral simethicon (80 mg; Sab Simplex gtt., Parke Davis GmbH, Berlin) was administered just before the investigation. Two hours after ingestion, the capsule position was verified. In cases of capsule persistence in the stomach, endoscopy-assisted insertion into the duodenal bulb was performed. Data were collected until the batteries discharged. Then the sensor array and data recorder were removed. The capsule endoscopy findings were evaluated by two endoscopists (I.T. and T.D.) experienced in small bowel investigation and blinded to the other data of the persons under investigation (including anamnestic, laboratory or clinical data). We evaluated the incidence and locations of small bowel injury including red spots and mucosal breaks (erosions or ulcers). A red spot was defined as a localised reddish area of mucosa without the loss of normal villous architecture, erosion as a tiny superficial destruction of mucosal surface with reddish or whitish base, aphthous lesion as a mucosal break with pale centre and a reddish halo, and ulcer as a larger excavated lesion with the base covered with fibrin (white) or hematin (black). All findings were classified into the three grades as mild enteropathy (multiple red spots, up to 10 erosions and aphthous lesions), moderate (10 to 20 erosions or aphthous lesions), and severe enteropathy (multiple erosions or aphthous lesions, more than 20, ulcers, stenosis, or bleeding). Isolated abnormal finding in the duodenal bulb was not considered to be an enteropathy. We used the method of rough estimation (using the time passed from the duodenal bulb) for capsule localization in the small bowel, because there are no clear endoscopy markers of borderlines between the jejunum and ileum (approximately one half comprises the jejunum and the other half the ileum). The small bowel visibility in different bowel segments was also evaluated.

### 2.2. Statistics

All data were compared between the groups with enteropathy and without enteropathy (identified by means of capsule endoscopy) by using the contingency tables and Fisher's exact test of independence, Armitage test for trend in proportions, and the chi-squared test for proportions in qualitative data. We used the two-sample *t*-test to compare two groups of qualitative data, and we also used the Mann-Whitney test whenever normality was not confirmed. Moreover, when the variances were distinct, we also applied the Kolmogorov-Smirnov test. When more than two groups were compared, we used the analysis of variance followed by the multiple comparison with the Fisher's LSD test. When normality was not confirmed, we applied the nonparametric analysis of variance, that is, the Kruskal-Wallis test followed by the multiple comparison with the Dunn test with Bonferroni modification. All the testing was performed on the five-percent significance level.

## 3. Results and Discussion

### 3.1. Endoscopy Findings

Wireless capsule endoscopy was successfully accomplished in all subjects. No complications of capsule endoscopy (including capsule retention) were observed during our study. Endoscopy-assisted insertion of capsule endoscope was organised due to the identification of capsule persistency in the stomach two hours after capsule ingestion in only two cases. The entire small bowel was recorded in 36 capsule endoscopies (97%). In one patient, the recording finished in the distal ileum. Visibility of the small bowel mucosa was focally decreased due to intestinal content in 17/37 cases (46%), mostly in the distal ileum. The mean gastric transit time of endoscopy capsule was 41 ± 41 minutes (median 30 minutes), small bowel transit time was 287 ± 107 minutes (median 274 minutes), and total time of investigation was 565 ± 67 minutes (median 553 minutes).

**Figure 1:**
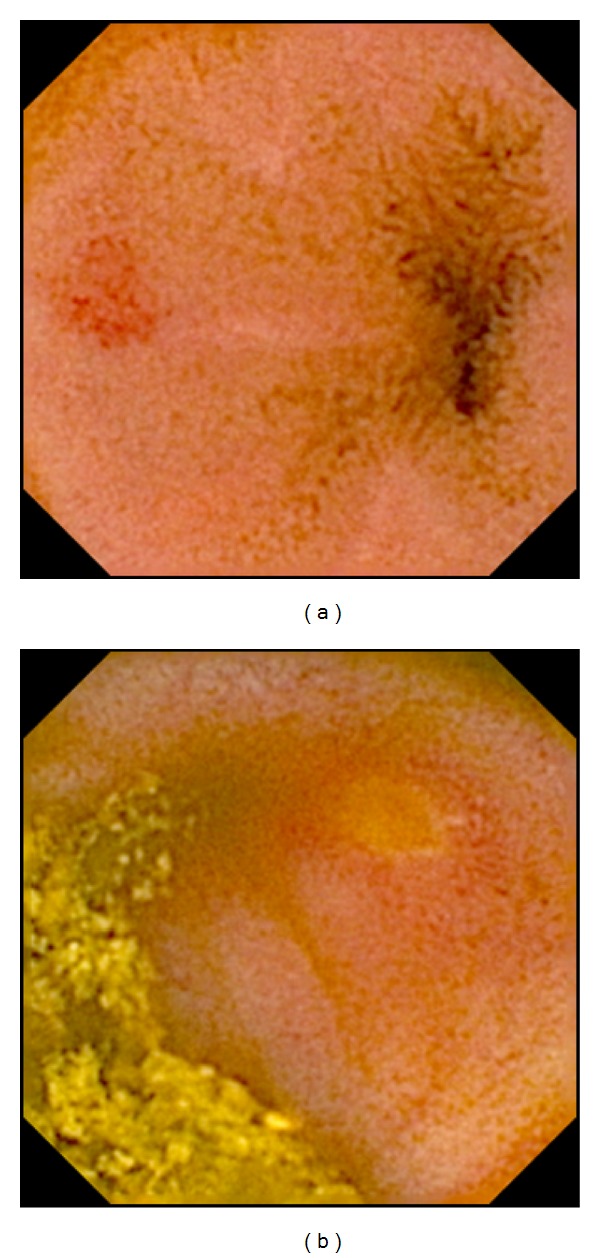
Mild NSAID-induced enteropathy. Red spot (a) and aphthous lesion (b).

**Figure 2:**
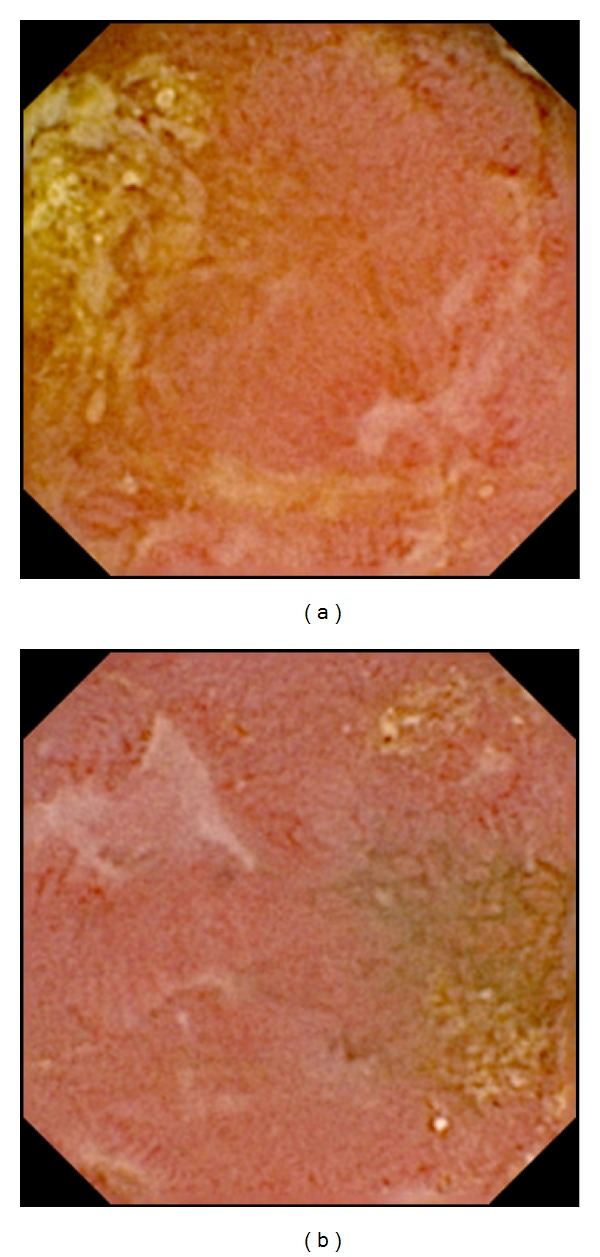
Moderate NSAID-induced enteropathy. Multiple erosions.

**Figure 3:**
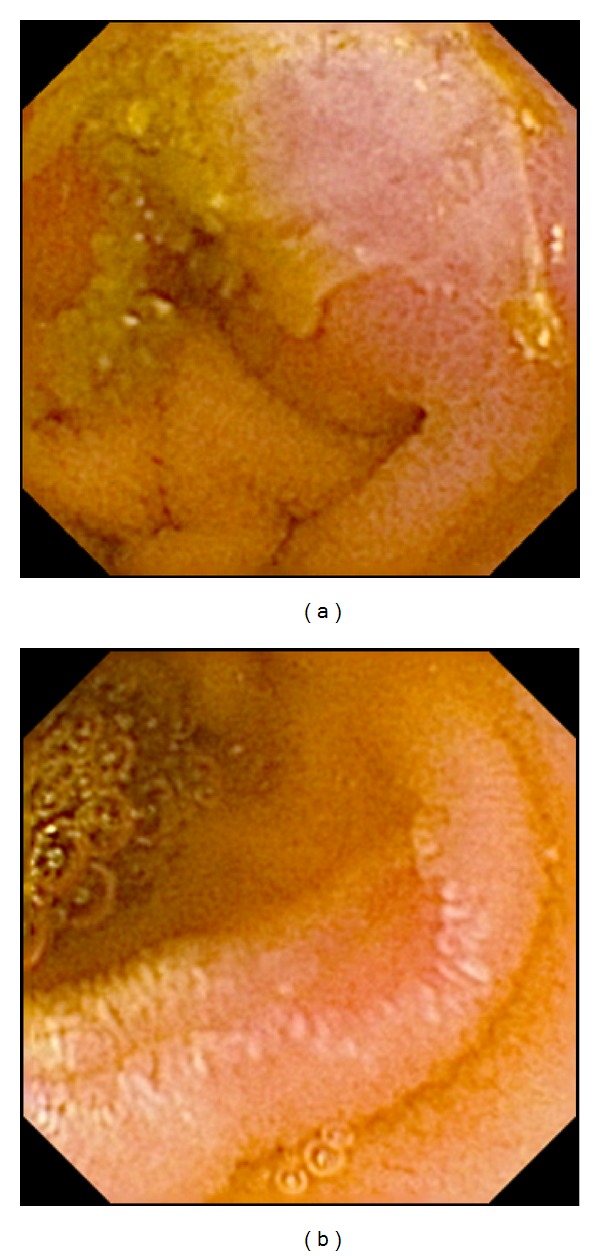
Severe NSAID-induced enteropathy, ulcers.

Abnormal endoscopy findings compatible with NSAID-induced enteropathy (Figures 1–3) were observed in 25/37 (68%) patients with rheumatoid arthritis (Table 1) and the enteroscopy picture was quite normal in 12/37 or evaluated as nonsignificant findings only (isolated red spots, lymphangiectasias, phlebectasias, xanthomas, and suspected lipoma). The enteropathy classification was mild in 18 (49%) investigated persons (Figure 1), with the most frequently described lesions being multiple red spots. Moderate enteropathy with multiple erosions and aphthous lesions (Figure 2) was observed in 4 (11%) and severe enteropathy with small intestinal ulcers (Figure 3) in 3 (8%) patients. The frequency of the lesions was similar in the jejunum and ileum (Table 2) No small bowel bleeding or stenoses were diagnosed in our patients. Abnormal findings outside the small bowel were described in 17 patients, mostly the endoscopy picture of erythematous or erosive gastritis.

The correlation between the presence of enteropathy and transit times was measured. No statistically important differences were observed in patients with and without enteropathy (*P* = 0.198 for gastric transit time and *P* = 0.794 for small bowel transit time).

### 3.2. Anamnestic Data

Rheumatoid arthritis was RF seropositive in 24 cases (65%) and was classified according the Steinbrocker's classification as grade I in 2, grade II in 13, grade III in 16, and grade IV in 6 patients. The average disease activity score (DAS 28) was 3.9 ± 1.5 (median 3.6). The mean duration of disease management was 13 years (median 8 years; first symptoms of disease at 42 years, median 44 years of age). All patients used NSAIDs: nonselective were used by 7 patients (19%; ibuprofen, diclofenac, ketoprofen, tiaprofenic acid, and oxaprozin), COX-2 preferential NSAIDs by 12 patients (32%; nimesulide and meloxicam), and COX-2 selective NSAIDs by 4 patients (11%; celecoxib). Most patients used NSAIDs for several years (more than 1 year, *n* = 33), and only 4 patients were treated by NSAIDs for less than one year.

Four persons (11%) used acidum acetylsalicylicum, 33 (89%) prednisone, 25 (68%) proton pump inhibitors (omeprazole), 9 (24%) bisfosfonates, 3 (8%) oral anticoagulants (warfarin), and 3 (8%) iron supplements. Dyspepsia (for our study defined as any type of abdominal discomfort including nausea, vomiting, abdominal fullness, pain, constipation, and diarrhoea) was present in 14 (38%) patients, and suspected signs of anaemic syndrome (weakness, dyspnoea, dizziness, and palpitation) were identified in 27 (73%) patients.

No correlation between the enteropathy and rheumatoid arthritis grade (according to the Steinbrocker, *P* = 0.382) or activity (*P* = 0.710) was observed. The difference between the average age of patients with NSAID-induced enteropathy (57 ± 16 years; median 56 years) and persons with normal small bowel endoscopy findings (51 ± 17 years; median 51 years) was not statistically significant (*P* = 0.297). No statistically significant difference in enteropathy presence according to gender was observed (*P* = 1.00).

### 3.3. Laboratory Tests

We focused on anaemia, nutritional status, and inflammatory markers in laboratory tests. A total of 25 patients displayed anaemia (mean haemoglobin level 107 ± 12.0 g/L, median 112 g/L) and 12 had positive FOBT. Thirteen (52%) had microcytic anaemia (mean corpuscular volume < 84 fL), 11 (44%) had normocytic anaemia (mean corpuscular volume 84–98 fL), and one had macrocytic anaemia (mean corpuscular volume >98 fL). Other results are given in Table 3. No statistically significant difference in haemoglobin level: *P* = 0.225, mean corpuscular volume: *P* = 0.266, haematocrit: *P* = 0.090, total erythrocyte count: *P* = 0.219, serum iron: *P* = 0.212 and binding capacity of iron: *P* = 0.212 between patients with enteropathy and normal small bowel findings was observed. There was no statistically significant difference in haemoccult positivity in patients with and without NSAID-induced enteropathy (*P* = 0.263).

Lower serum albumin level (<35 g/L) was present in one subject with rheumatoid arthritis only, lower serum prealbumin (<0.2 g/L) levels in seven persons. Statistically significant differences between patients with normal enteroscopy findings and those with enteropathy-compatible findings in serum albumin (*P* = 0.824) and/or prealbumin (*P* = 0.127) levels were not proved. Other results are given in Table 4.

The mean serum CRP level was 18.1 ± 22.4 mg/L (median 7.0), and elevated CRP (6–68 mg/L) was found in 20 subjects (54%). Leukocytosis (9.4 × 10^9^/L) was found in 16 individuals (43%), the mean leukocyte level was 9.2 ± 2.9 × 10^9^/L (median 8.6 × 10^9^/L), and accelerated erythrocyte sedimentation rate was found in 19 patients (after 1 hour). No statistically significant difference in inflammatory markers was observed between subjects with and without enteropathy (CRP: *P* = 0.320; leukocytes level: *P* = 0.912; and erythrocyte sedimentation rate after 1 hours: *P* = 0.183). Other results are given in Table 5.

### 3.4. Discussion

The aim of our study was to evaluate the basic characteristics (endoscopic, laboratory, and clinical) of NSAID-induced enteropathy as well as the diagnostic yield of wireless capsule endoscopy.

Capsule endoscopy confirmed its safety in the subgroup of patients with rheumatoid arthritis and occult gastrointestinal bleeding. Although some data identified NSAIDs as a possible risk factor for capsule retention [24], we observed no significant complication including capsule nonpassage in our study. Limited visibility was present in 46% of all patients, capsule endoscopy was performed after fasting, and bowel preparation would probably reduce this problem. On the other hand, bowel content was localised only focally in most cases, so the majority of mucosal surface was visible.

The overall prevalence of enteropathy was 68% in the study population. The observed prevalence is fully comparable with studies using noninvasive tests in NSAID-induced enteropathy [25]. On the other hand, the prevalence is much higher than previously estimated by earlier surgical or autopsy studies [15, 26]. Our observations further confirm the high sensitivity of capsule endoscopy and possibility of detection of subclinical small bowel damage. About half of all subjects had mild enteropathy with multiple red spots and some erosions (or aphthous lesions) presented in low numbers (up to 10). The clinical importance of abnormal findings like this is extremely controversial.

Another problem that complicates interpretation of so-called mild enteropathy (especially the red spots findings) is a repeatedly confirmed occurrence of similar abnormalities in the small intestine of healthy volunteers or in the control groups treated with placebo (7–41%) in previously published capsule endoscopy studies [17–21]. To minimize the risk of misinterpretation of red spots findings, only multiple lesions were described as enteropathy (a single red spot was considered normal finding). Severe damage of small intestinal mucosa (ulcers) was present in 19% of long-term NSAIDs users.

The lesions identified in our patients (erythema, erosions, aphthous lesions, and ulcers) can be a relatively nonspecific reaction of small bowel mucosa to damage or inflammation of a different aetiology. Marking of these findings as NSAID-induced enteropathy is possible only after exclusion of other causes on the basis of total endoscopic image in combination with other laboratory, clinical, and anamnestic data. Crohn's disease, infectious and parasitic diseases, coeliac disease and its complications, vasculitis, and Behcet's syndrome were excluded in all our patients with small bowel lesions.

The relationship between NSAID-induced enteropathy and occult gastrointestinal bleeding was demonstrated (especially by means of radioisotope-labelled erythrocytes) already in the 1980s [27]. We tried to prove the predictive value of microcytic iron-deficiency anaemia and/or positive FOBT for presence of NSAID-induced enteropathy by correlation of laboratory and endoscopy data together. Although capsule endoscopy examination was performed in a preselected population of patients with rheumatoid arthritis and anaemia and/or positive FOBT, the prevalence was similar to the data presented in nonselected population of NSAIDs users [18–20, 28, 29]. Following statistical analysis we found no significant correlations between the presence/absence of enteropathy and anaemia markers (haemoglobin level, mean corpuscular volume, haematocrit, erythrocyte count, serum iron, and binding capacity of transferrin) and FOBT positivity. There can be several explanations. Erosions (or aphthous lesions) often lead to increased blood loss in the gastrointestinal tract, but the low intensity is not sufficient to induce laboratory abnormalities. Aetiology of anaemia is in patients with NSAID enteropathy often combined with disturbances in absorption of iron and vitamin B12 modifying the character of anaemia [30]. The anaemia of chronic illness in patients with rheumatoid arthritis should be also considered. Another problem is the limited specificity of the guaiac-based FOBT used. These facts indicate the limited value of anaemia and FOBT as predictors of small bowel damage in NSAID users with rheumatoid arthritis.

The correlation of nutritional (albumin, prealbumin) and inflammatory markers (CRP, leukocytes, and erythrocyte sedimentation rate) and NSAID-induced enteropathy was statistically evaluated, too. Small loss of proteins through the damaged small intestinal mucosa, persisting for a longer time after NSAIDs discontinuation, is found frequently [5]. The aetiology of hypoalbuminaemia in rheumatoid arthritis patients (up to the 10%) is wide, including secondary protein loosing enteropathy, amyloidosis, and rheumatoid arthritis itself. Therefore these markers cannot be an ideal predictor of the presence of NSAID-induced enteropathy (our data supporting this assumption, no clinically significant correlation was observed between the presence/absence of enteropathy and albumin/prealbumin levels). The basic inflammatory markers (CRP, leukocytes, and erythrocytes sedimentation) are not applicable due to their relative nonspecificity for NSAID-induced enteropathy in subjects with rheumatoid arthritis. Important limitation of our study is the absence of recent colonoscopy findings in most patients. Correlation of laboratory markers and enteroscopy findings may be distorted by the presence of undiagnosed NSAID-induced colopathy. Colonoscopy was performed in 8 of 12 patients with positive FOBT; 3 of them displayed abnormal findings inside the large bowel (multiple adenomatous polyps, diverticula, and angiectasias).

Clinical symptoms (dyspepsia broadly defined as any abdominal discomfort including pain, diarrhea, and constipation) seem to be unusable for selection of patients with suspected small bowel damage induced by NSAIDs. Infrequent studies similarly show a weak association between dyspepsia and small intestinal lesions (ulcerations of the jejunum and ileum can cause noncharacteristic dyspepsia and abdominal pain). Stenosis can be presented with subsequent passage disorders [12, 31, 32]. On the other hand, the direct effect of NSAIDs on the central nervous system can also induce nonspecific dyspepsia [33]. Another problem confounding results may be the relatively high frequency of dyspepsia in the general population in the Czech Republic (17%) [34]. 

The lower prevalence of small intestinal damage in experimental animals or patients using COX-2 selective or preferential NSAIDs compared with nonselective COX drugs has been demonstrated repeatedly [20, 28, 35, 36]. In capsule endoscopy studies, this difference was statistically significant [18, 20] or insignificant but still present in most cases [37]. The spectrum of NSAIDs used in our patients confirms the current trend towards leaving traditional nonselective NSAIDs and potentially cardiotoxic COX-2 selective NSAIDs for COX-2 preferential drugs. Enteropathy findings were observed in 6 patients treated with nonselective NSAIDs (67% of nonselective NSAIDs users), 17 patients treated with COX-2 preferential drugs (74% of COX-2 preferential users), and 2 patients treated with COX-2 selective drugs (40% of COX-2 selective users). The difference between the groups was not statistically significant, but a trend is noticeable: lower frequency of enteropathy in patients treated using COX-2 selective drugs. Another trend observed is downward severity of the lesions in relation to the higher COX-2 selectivity. The occurrence of mucosal breaks may be observed in patients taking COX nonselective NSAIDs. Mucosal breaks were found in patients taking COX-2 selective agent in our group in 1 of the 6 patients only (17%). It must be emphasised that long-term treatment using COX-2 selective NSAIDs also leads to damage of the small intestine, although probably at a lower frequency and severity.

## 4. Conclusions 

In conclusion, NSAID therapy causes small bowel lesions in a significant section of rheumatoid arthritis patients that equals (or even exceeds) the frequency of this disorder observed in the upper part of the digestive tract (in the oesophagus, stomach and duodenum). Although the clinical importance of NSAID-induced enteropathy is often limited, it can lead to severe complications. Capsule endoscopy has become sensitive diagnostic method in identifying of mucosal breaks or other types of small bowel lesions. We also focused on laboratory and/or clinical predictors for NSAID-induced enteropathy. Anaemia, nutrition (albumin, prealbumin), and inflammatory markers (CRP, thrombocytosis, and erythrocyte sedimentation rate) cannot be recommended for diagnostics of NSAID-induced enteropathy. The clinical factors (gender, dyspepsia) appeared not to be very reliable too.

## Figures and Tables

**Table 1:** Abnormal findings compatible with NSAID-induced lesions (MDS-mucosal damage score): 1 mild enteropathy, 2 moderate enteropathy, and 3 severe enteropathy.

	Sex	Age	Duodenum	Jejunum	Ileum	Other localisation	MDS
(1)	M	50		3 linear erosions, red spot		Gastric erosions	1
(2)	F	66	Red spots	Red spots	Red spots	Erythematous gastritis	1
(3)	F	35	Multiple red spots	Erosion, 7 aphthous lesions	10 aphthous lesions		2
(4)	F	53	Red spot				0
(5)	F	78	Multiple erosions	Erosions, aphthous lesions, ulcers	Erosions, aphthous lesions, ulcers		3
(6)	F	28			Single red spot		0
(7)	F	72		Red spots, multiple aphthous lesions		Erythematous gastritis	2
(8)	F	61		Red spots		Erythematous gastritis	1
(9)	F	49			Single red spot		0
(10)	M	62			3 erosions	Erythematous gastritis	1
(11)	F	32			Single red spot		0
(12)	F	69		Erosions, multiple aphthous lesions	Single erosion	Gastric ulcer	2
(13)	F	82		Red spots, erosions			1
(14)	M	56		Red spots, erosions			1
(15)	F	34	Bulbitis	Single red spot	Single red spot, single erosion	Gastric erosions	1
(16)	F	42				Gastric focal erythema	0
(17)	F	71		Single erosion	8 erosions	Erythematous gastritis	1
(18)	M	57					0
(19)	F	79			Single red spot		0
(20)	F	38		Single erosion	4 erosions		1
(21)	F	26				Gastric erosions	0
(22)	F	69		Single erosion	5 erosions		1
(23)	M	55			Red spots		1
(24)	F	22	Red spots	Single erosion	5 erosions	Gastric oedema	1
(25)	F	73		Red spot		Erythematous gastritis	0
(26)	F	76			Single erosion		1
(27)	F	55		Single erosion	5 erosions		1
(28)	F	56		Ulcer	Ulcer		3
(29)	F	43					0
(30)	F	56		2 erosions			1
(31)	M	60	Bulbitis			Erythematous gastritis	0
(32)	F	64					0
(33)	F	54		Single erosion	Ulcer		3
(34)	M	56		Single erosion	Multiple erosions, aphthous lesion	Gastric erosions	2
(35)	F	61			Erosion, aphthous lesions		1
(36)	F	23			Red spots	Gastric oedema	1
(37)	F	57		Single erosion	Red spots, aphthous lesion		1

**Table 2:** Distribution of small intestinal lesions.

Localisation of findings	Enteropathy severity (1: mild, 2: moderate, 3: severe)		
	1, 2 and 3	2 and 3	3
	*N = 42	*N* = 14	*N* = 6
Duodenum	4	1	0
Jejunum	19	7	3
Ileum	19	6	3

*Number of abnormal findings compatible with enteropathy.

**Table 3:** Anaemia markers in patients with rheumatoid arthritis according to NSAID-induced enteropathy.

	Mucosal damage score	*N*	Mean	SD	Median	1st quartile	3rd quartile	Normality
Hb (g/L)	No enteropathy	12	109.0	15.9	111.5	100.5	116.5	n
	Any enteropathy	25	116.0	15.4	115.0	105.5	126.0	n
	1	18	119.0	14.2	115.0	110.3	129.5	n
	2	4	113.0	11.5	110.0	103.0	124.5	n
	3	3	101.0	21.2	109.0	77.0	117.0	n

MCV (fL)	No enteropathy	12	85.0	12.0	83.8	74.2	91.8	n
	Any enteropathy	25	88.1	6.9	87.4	81.5	94.2	n
	1	18	89.8	6.4	90.4	86.4	94.7	n
	2	4	84.2	9.1	80.9	78.1	93.7	n
	3	3	82.7	2.1	81.8	81.2	85.1	n

Htk	No enteropathy	12	0.333	0.037	0.332	0.303	0.362	n
	Any enteropathy	25	0.357	0.040	0.355	0.331	0.387	n
	1	18	0.365	0.039	0.365	0.333	0.399	n
	2	4	0.351	0.025	0.343	0.334	0.378	n
	3	3	0.321	0.057	0.324	0.263	0.377	n

Ery (×10^12^/L)	No enteropathy	12	4.22	0.53	4.26	4.05	4.50	n
	Any enteropathy	25	4.01	0.46	3.98	3.78	4.43	n
	1	18	3.99	0.48	3.94	3.73	4.45	n
	2	4	4.19	0.22	4.15	3.99	4.41	n
	3	3	3.88	0.60	3.96	3.24	4.43	n

Fe (*μ*mol/L)	No enteropathy	12	10.0	8.2	6.7	3.6	15.0	nn
	Any enteropathy	25	11.8	5.8	11.3	7.8	15.4	n
	1	18	12.4	5.7	13.4	8.5	15.7	n
	2	4	9.0	5.4	8.6	4.0	14.4	n
	3	3	12.0	8.6	10.5	4.3	21.2	n

VK (*μ*mol/L)	No enteropathy	12	63.1	12.5	64.7	51.7	73.2	n
	Any enteropathy	25	56.4	14.5	57.7	52.1	65.6	nn
	1	18	55.0	15.6	56.5	50.7	66.9	nn
	2	4	62.0	14.8	60.5	49.0	76.5	n
	3	3	57.3	6.3	55.1	52.3	64.4	n

Mucosal damage score: 1 mild enteropathy, 2 moderate enteropathy, 3 severe enteropathy, Hb (g/L): haemoglobin level,MCV (fL): mean corpuscular volume,Htk: haematocrit,Ery (×10^12^/L): erythrocytes count, Fe (*μ*mol/L): serum iron concentration, and BK (*μ*mol/L): binding capacity of transferrin. Normality of data distribution: normal (n) or nonnormal (nn).

**Table 4:** Nutrition markers in patients with rheumatoid arthritis according to NSAID-induced enteropathy.

	Mucosal damage score	*N*	Mean	SD	Median	1st quartile	3rd quartile	Normality
Alb (g/L)	No enteropathy	12	41.9	3.9	42.8	37.7	44.7	n
	Any enteropathy	25	42.2	3.6	41.6	40.2	44.7	n
	1	18	42.6	3.9	42.9	39.9	45.1	n
	2	4	42.4	2.7	41.5	40.6	45.1	n
	3	3	39.5	2.4	40.4	36.8	41.4	n

Palb (g/L)	No enteropathy	12	0.25	0.11	0.23	0.19	0.27	nn
	Any enteropathy	25	0.28	0.090	0.25	0.22	0.31	nn
	1	18	0.30	0.096	0.29	0.23	0.34	nn
	2	4	0.22	0.029	0.23	0.19	0.25	n
	3	3	0.22	0.046	0.25	0.17	0.25	nn

Mucosal damage score: 1 mild enteropathy, 2 moderate enteropathy, 3 severe enteropathy, Alb (g/L): albumin level, and Palb (g/L): prealbumin level. Normality of data distribution: normal (n) or nonnormal (nn).

**Table 5:** Inflammatory markers in patients with rheumatoid arthritis according to NSAID-induced enteropathy.

	Mucosal damage score	*N *	Mean	SD	Median	1st quartile	3rd quartile	Normality
CRP (mg/L)	No enteropathy	12	21.0	21.8	12.5	2.0	40.5	nn
	Any enteropathy	25	17.0	23.0	5.0	1.0	34.0	nn
	1	18	10.0	14.4	3.0	1.0	11.0	nn
	2	4	19.0	33.1	3.0	0.3	52.3	nn
	3	3	57.0	12.1	61.0	43.0	66.0	n

Leu (×10^9^/L)	No enteropathy	12	9.3	3.0	9.9	7.2	11.4	n
	Any enteropathy	25	9.2	2.9	8.6	7.2	11.4	n
	1	18	9.8	3.2	9.2	77.9	12.6	n
	2	4	7.5	1.3	7.4	6.3	8.9	n
	3	3	8.1	1.5	7.5	6.8	9.6	n

ESR1	No enteropathy	12	23.0	21.2	15.5	7.0	25.5	nn
	Any enteropathy	25	34.0	24.6	26.0	13.0	51.5	nn
	1	18	32.0	25.9	23.0	9.5	51.8	nn
	2	4	27.0	16.3	24.5	13.0	42.8	n
	3	3	52.0	24.0	52.0	28.0	76.0	n

ESR2	No enteropathy	12	41.0	27.4	32.5	18.5	51.3	n
	Any enteropathy	25	54.0	30.6	52.0	29.0	78.0	n
	1	18	52.0	31.7	53.0	21.8	78.5	n
	2	4	46.0	21.6	40.5	29.0	68.5	n
	3	3	79.0	30.5	80.0	48.0	109.0	n

Mucosal damage score: 1 mild enteropathy, 2 moderate enteropathy, 3 severe enteropathy, CRP (mg/L): C-reactive protein, Leu (×10^9^/L): leukocytes count, and ESR: sedimentation of erythrocytes after 1 and 2 hours. Normality of data distribution: normal (n) or nonnormal (nn).
